# Machine Learning in Stem Cell Research: From Biological Data to Clinical Translation

**DOI:** 10.34133/csbj.0212

**Published:** 2026-09-30

**Authors:** Anna Nicolaou, Yan Hong, Delong Zhou, Geovanni Geukgeuzian, Vasisht Yegneshwaran, Nehal Kamal Ali, Diego Fraidenraich, Xueqing Huang

**Affiliations:** ^1^ Department of Computer Science, New York Institute of Technology, Old Westbury, NY 11568, USA.; ^2^ Department of Cell Biology & Molecular Medicine, Rutgers New Jersey Medical School, Newark, NJ 07101, USA.

## Abstract

Stem cell (SC) research plays a central role in disease modeling and regenerative medicine, yet its clinical translation remains challenged by biological heterogeneity, incomplete cellular maturation, stringent quality-control requirements, and increasingly complex biological and clinical data. Machine learning (ML) has emerged as a powerful computational approach for extracting biologically meaningful information from these data, enabling quantitative characterization of SC behavior and predictive modeling across diverse experimental and translational applications. This review presents a unified framework for ML in SC research by organizing the literature according to SC data sources and representations, ML methodologies and model families, and biological and clinical application domains. We review ML applications spanning SC reprogramming, differentiation, postdifferentiation maturation, quality control, disease modeling, therapeutic development, and clinical decision support, highlighting how diverse experimental data, including molecular and cellular profiling, biomedical imaging, biochemical profiling, functional measurements, and clinical data, enable biological interpretation, predictive modeling, and translational decision-making. We further discuss benchmark dataset selection, validation strategies, transformer-based foundation models, and emerging multimodal learning approaches and propose benchmark dataset selection criteria emphasizing biological diversity, experimental rigor, metadata completeness, reproducibility, and public accessibility. Collectively, this review provides a comprehensive framework for understanding current ML applications in SC research and outlines future directions toward robust, interpretable, and clinically translatable computational frameworks.

## Introduction

The rapid growth of biomedical data, together with advances in machine learning (ML), has transformed biomedical research by enabling quantitative analysis of complex biological systems and facilitating predictive modeling and decision support [1]. One area in which these advances have had increasing impact is stem cell (SC) research [2,3]. Through self-renewal and lineage-specific differentiation, SCs contribute to tissue development, regeneration, and repair, making them fundamental to disease modeling, drug discovery, regenerative medicine, and precision medicine [4]. SC research generates large volumes of heterogeneous data from diverse experimental sources. The complexity, high dimensionality, biological variability, and multiple data representations of these data present substantial analytical challenges for conventional computational approaches. ML helps address these challenges by learning informative representations, identifying biological patterns, discovering biomarkers, and developing predictive models for the quantitative characterization of SC behavior.

Recent advances in deep learning, particularly transformer-based architectures and foundation models, have further expanded ML capabilities through large-scale pretraining, transfer learning, and representation learning [5–7]. These models learn generalizable biological representations that can be adapted to diverse downstream tasks such as cell type annotation, perturbation prediction, multi-omics integration, drug-response prediction, and biological knowledge discovery. While their adoption in SC research remains at an early stage, foundation models offer considerable potential for integrating heterogeneous SC data, improving generalization across datasets, and enabling more robust and scalable predictive modeling.

Several reviews have summarized ML applications in SC research [1–3]. However, most focus on specific applications or ML techniques rather than providing a unified framework that connects SC data, computational methodologies, biological discovery, and clinical translation. Representation-specific preprocessing, benchmark dataset development, validation strategies, and emerging approaches such as foundation models and multimodal learning have also received comparatively limited attention.

To address these gaps, this narrative review presents a unified framework for ML in SC research. We first introduce SC biology, SC data, and the computational methodologies that underpin ML before reviewing 2 major research domains: (a) SC biology and engineering and (b) clinical applications. Finally, we discuss current challenges and future directions for clinically translatable ML. The key contributions of this review are summarized as follows:

• proposing a unified framework that organizes ML in SC research according to SC data types, experimental data sources, data representations, computational methodologies, biological research domains, and clinical applications
• reviewing preprocessing strategies, learning paradigms, ML model families, model selection, and validation methodologies for heterogeneous SC data
• synthesizing ML advances across SC biology and engineering (reprogramming, differentiation, postdifferentiation maturation, and quality control) and clinical applications (disease modeling, therapeutic development, and clinical decision support)
• identifying future opportunities in benchmark dataset development, multimodal learning, foundation models, interpretability, external validation, and clinically translatable ML

The remainder of this paper is organized as follows: Concepts of SC Biology and Applications introduces SC biology and its major applications. SC Data Types, Sources, and Representations and Statistical and ML Models in SC Research review SC data and the computational methodologies that underpin ML in SC research. Research Domain I: SC Biology and Engineering and Research Domain II: Clinical Applications present SC biology and engineering and clinical applications, respectively. Discussion and Future Directions discusses current challenges and future research directions, and Conclusion concludes the paper.

## Concepts of SC Biology and Applications

This section introduces the principal SC categories and their major biomedical applications. Cellular potency, lineage commitment, differentiation, and maturation provide the biological foundation for the ML methodologies and application domains discussed in subsequent sections. Key cell types and ML-method abbreviations are summarized in Tables 1 and 2, respectively.

**Table 1.** Abbreviations used for stem-cell-related cell types

Cell group	Abbreviation	Full name
Pluripotent stem cells	PSC	Pluripotent stem cell
	ESC	Embryonic stem cell
	iPSC	Induced pluripotent stem cell
Hematopoietic stem/progenitor cells	HSPC	Hematopoietic stem and progenitor cell
	HSC	Hematopoietic stem cell
	LT-HSC	Long-term hematopoietic stem cell
	ST-HSC	Short-term hematopoietic stem cell
	MPP	Multipotent progenitor cell
Tissue-specific stem/progenitor cells	MSC	Mesenchymal stem/stromal cell
	SHED	Stem cells from human exfoliated deciduous teeth
	MuSC	Muscle stem cell
	NSC	Neural stem cell
	NPC	Neural progenitor cell
	CPC	Cardiac progenitor cell
	RPESC	Retinal pigment epithelial stem cell
Differentiated cells	CM	Cardiomyocyte
	RPE	Retinal pigment epithelial cell
Species-specific cells	mESC	Mouse embryonic stem cell
	mPSC	Mouse pluripotent stem cell
	miPSC	Mouse induced pluripotent stem cell
	mHSC	Mouse hematopoietic stem cell
	mMPP	Mouse multipotent progenitor cell
	hPSC	Human pluripotent stem cell
	hiPSC	Human induced pluripotent stem cell
	hESC	Human embryonic stem cell
	hMSC	Human mesenchymal stem/stromal cell
	hBMSC	Human bone marrow mesenchymal stem/stromal cell
	hNTSC	Human neural-crest-derived nasal turbinate stem cell
Supporting cells	MEF	Mouse embryonic fibroblast
	PBMC	Peripheral blood mononuclear cell
Cancer stem cells	CSC	Cancer stem cell
	LSC	Leukemic stem cell

**Table 2.** Abbreviations used for machine learning methods in stem cell research

Abbreviation	Full name/description
Feature engineering and dimensionality reduction	
PCA	Principal component analysis
LASSO	Least absolute shrinkage and selection operator
l_1_l_2FS_	Elastic-Net feature selection method
RFE	Recursive feature elimination
MCFS	Monte Carlo feature selection
FFT	Fast Fourier transform
SIFT	Scale-invariant feature transform
t-SNE	t-distributed stochastic neighbor embedding
UMAP	Uniform manifold approximation and projection
Classical machine learning	
→ Linear, kernel-based, and instance-based models	
LR	Logistic regression
OCLR	One-class logistic regression
LDA	Linear discriminant analysis
AROW	Adaptive regularization of weights
SVM	Support vector machine
RBF-SVM	Radial basis function support vector machine
SVR	Support vector regression
KNN	*k*-nearest neighbors (e.g., 1-NN)
→ Tree-based and ensemble models	
DT	Decision tree
ADT	Alternating decision tree
BDT	Boosted decision tree
RF	Random forest
TreeBagger	MATLAB bagged decision tree ensemble
SGB	Stochastic gradient boosting
XGBoost	Extreme gradient boosting
→ Unsupervised learning	
*K*-means	*K*-means clustering
GMM	Gaussian mixture model
AGA	Automatic gating algorithm
Deep learning	
→ Convolutional and recurrent neural networks	
(A)NN	(Artificial) Neural network
MLP	Multilayer perceptron
DNN	Deep neural network
CNN	Convolutional neural network
RNN	Recurrent neural network
LSTM	Long short-term memory
BLSTM	Bidirectional long short-term memory
Att-BLSTM	Attention-based bidirectional long short-term memory
→ Generative models	
VQ-VAE	Vector quantized variational autoencoder
GAN	Generative adversarial network
(c)GAN	(Conditional) Generative adversarial network
pix2pix	Conditional GAN for image-to-image translation
→ Transformer-based and foundation models	
ViT	Vision transformer
GPT	Generative pretrained transformer
Geneformer	Foundation model for single-cell transcriptomics
scGPT	Single-Cell GPT
scFoundation	Single-Cell Foundation Model
BioMedGPT	Biomedical Multimodal GPT
Probabilistic, statistical, and rule-based models	
NB	Naïve Bayes
TAN	Tree-augmented naïve Bayes
BN	Bayesian network
HMM	Hidden Markov model
Cox	Cox proportional hazards model
BNMA	Bayesian network meta-analysis
CARM	Classification association rule mining
Optimization and interpretability	
SAGA	Stochastic average gradient augmented
Grad-CAM	Gradient-weighted class activation mapping
Application-specific models and pipelines	
SteMClass	DNA methylation-based iPSC differentiation classifier
hiPSCore	Machine-learning-based hiPSC quality classifier
MeD-P	Mesenchymal Stem Cell Differentiation Prediction
OSCORP	Optimized digital cancer stem cell sorter and profiler
Dual-FLIT	Dual fluorescence imaging time lapse
PhenoLOGIC	High-content image analysis software
DREDDA	Drug Repositioning through Expression Data Domain Adaptation
DERGA	Data Ensemble Refinement Greedy Algorithm
LSM	Deep-learning-based HSC classification model

### Overview of SC types

SCs are commonly classified according to developmental potency, or their capacity to differentiate into specialized cell types (Fig. 1). The 2 principal categories are pluripotent stem cells (PSCs), including embryonic stem cells (ESCs) and induced pluripotent stem cells (iPSCs), and multipotent stem cells (MPSCs) [8]. PSCs exhibit broad differentiation potential and self-renewal capacity, making them valuable for developmental studies, disease modeling, therapeutic discovery, and regenerative medicine.

**Fig. 1.**
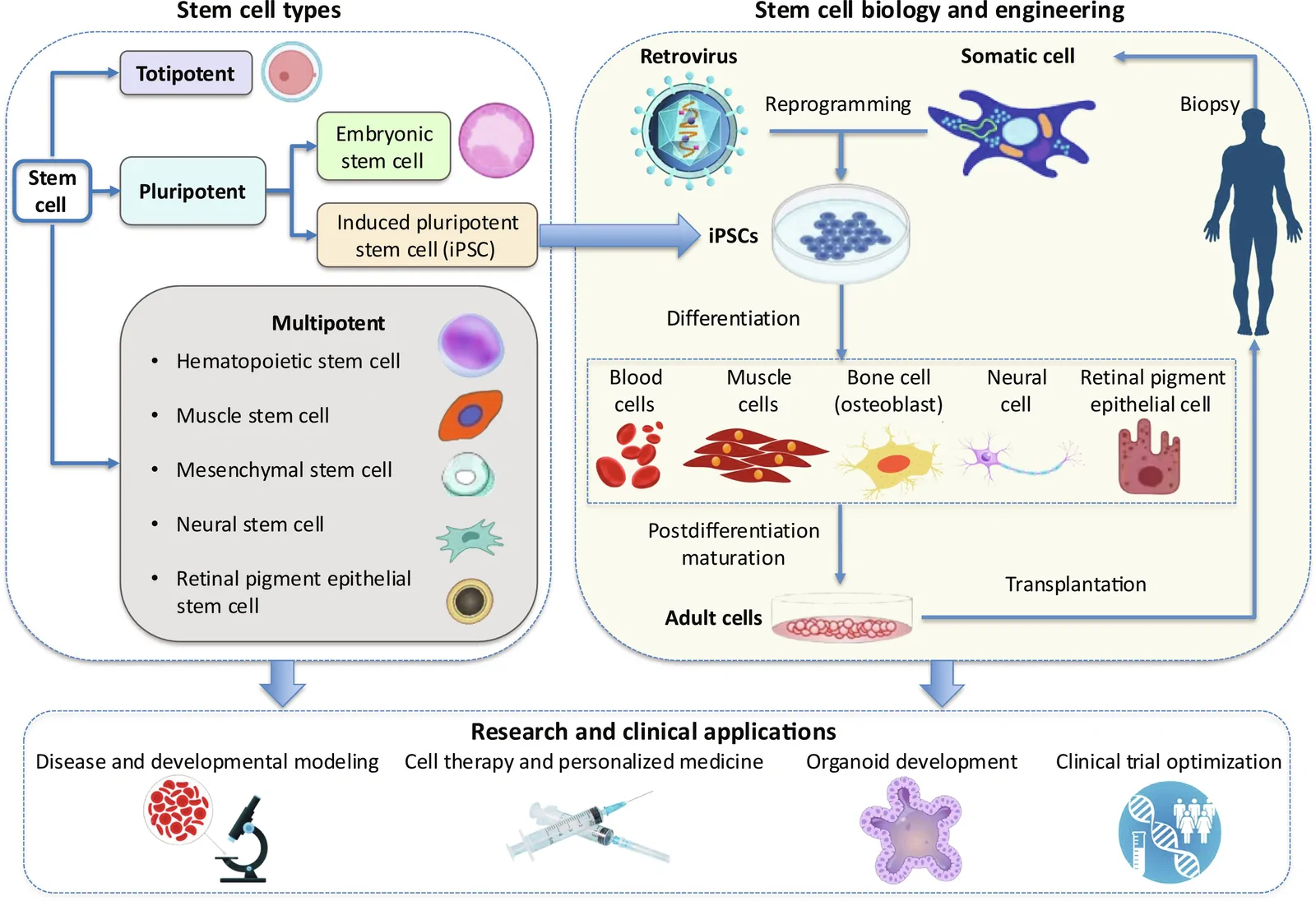
Overview of stem cell types, stem cell biology and engineering, and representative research and clinical applications.

ESCs remain an important model for studying early development because of their stable pluripotency and broad differentiation potential [9]. However, ethical considerations and species-specific differences between animal and human ESCs have limited their broader application. iPSCs overcome many ethical concerns associated with ESCs by reprogramming somatic cells into an ESC-like state through ectopic expression of Yamanaka transcription factors [10], which induce and maintain pluripotency. Their patient-specific origin enables individualized disease modeling and supports personalized therapeutic development and regenerative medicine. Nevertheless, iPSC-based approaches remain constrained by variable reprogramming efficiency, incomplete differentiation, genomic instability, and stringent quality-control requirements.

Compared with PSCs, MPSCs exhibit more restricted differentiation potential but generally present lower tumorigenic risk and have seen broader clinical application. Nevertheless, their therapeutic utility still depends on careful control of culture conditions to preserve lineage commitment and functional properties.

### SC applications

Following directed differentiation and functional maturation, SCs enable a broad range of biomedical applications spanning basic research, therapeutic development, and clinical translation. These applications require scalable cell production, rigorous quality control, and reliable assessment of cellular identity, potency, and function.

#### Differentiation

Directed differentiation is fundamental to development, tissue homeostasis, and regeneration and can be regulated through biochemical, mechanical, and epigenetic cues [11]. ML integrates these interacting biological factors to model differentiation trajectories, predict lineage commitment, and identify determinants of lineage specification.

#### Postdifferentiation maturation

Postdifferentiation maturation is a gradual process through which differentiated cells acquire adultlike structural, biochemical, and functional properties required for transplantation, disease modeling, and drug testing [12]. Its assessment is complicated by continuous developmental trajectories and the absence of standardized maturity criteria. ML enables quantitative assessment of maturation state, estimation of developmental progression, and identification of biomarkers associated with cellular maturity.

#### Quality control and production

Quality control is essential for maintaining differentiation efficiency, scalability, cellular function, and therapeutic suitability [13]. ML supports scalable manufacturing by enabling automated monitoring of cell quality, potency, maturation state, and production consistency while reducing manual assessment and improving process reproducibility.

#### Disease modeling and therapeutic development

Patient-derived SC models provide physiologically relevant platforms for investigating disease mechanisms, evaluating therapeutic responses, and advancing regenerative medicine under controlled laboratory conditions. By preserving patient-specific genetic backgrounds, they enable the study of disease heterogeneity and facilitate personalized therapeutic strategies [14]. The subsequent sections review how ML enhances these applications through quantitative analysis, predictive modeling, and integration of heterogeneous biological and clinical data.

## SC Data Types, Sources, and Representations

This section distinguishes 3 related concepts used throughout this review: SC data types, experimental data sources, and data representations. SC data types refer to the biological or contextual information captured in SC research; experimental data sources refer to the technologies, assays, instruments, or clinical resources from which these data originate; and data representations refer to the form in which the information is organized for ML analysis.

### SC data types

SC studies generate heterogeneous data that capture distinct aspects of cellular identity, morphology, physiological function, and experimental context. These data characterize SC state and behavior and are acquired from the experimental data sources described in the “SC data sources” section. The discussion progresses from molecular information (transcriptomic and epigenomic data) to observable phenotype (morphological data), physiological activity (biochemical and functional data), and finally the experimental context provided by experimental process metadata. Representative ML input features for each data type are provided in Table 3. Together, these complementary data support biological interpretation, predictive modeling, and clinical translation.

**Table 3.** Representative machine learning input features, definitions, and data representations associated with major stem cell data types

Data type	Representative feature	Definition	Representation
Transcriptomic and epigenomic data	Normalized expression value [37,66]	Gene or transcript abundance normalized for technical variation	Tabular
	DNA methylation level [56,67]	Degree of DNA methylation within a genomic region	
Morphological data	Area [59,72]	Region occupied by a cell, a colony, or a cellular aggregate	Image, video; tabular
	Perimeter [59,72]	Length of the boundary of a segmented cell or colony	
Biochemical data	Metabolite abundance [48,80]	Relative or absolute abundance of metabolites	Tabular
	Protein biomarker level [121]	Quantitative abundance of protein biomarkers measured in plasma	
Functional data	Beat rate [87]	Frequency of spontaneous or electrically paced cellular contractions	Sequential; tabular
	Calcium-transient duration [44,104,105]	Duration of an intracellular calcium transient	
Experimental metadata	Cell line [59,68]	Identifier of the stem cell line or clone	Tabular (categorical)
	Donor characteristics [81]	Donor-associated attributes (e.g., age, sex, and tissue source)	
	Culture duration [81,82]	Duration for which cells are maintained under specified culture conditions	Tabular

#### Transcriptomic and epigenomic data

Gene expression and epigenomic profiling provide molecular information about cellular identity, differentiation, development, and disease. Transcriptomic data quantify transcript abundance, whereas epigenomic data describe regulatory mechanisms, such as DNA methylation, that influence gene expression without altering the underlying DNA sequence. For ML analysis, these data are typically represented as high-dimensional tabular matrices in which rows correspond to biological samples (or individual cells) and columns correspond to genes, transcripts, genomic loci, or other molecular features. Representative features include read counts, normalized expression values, methylation levels, and log fold change.

#### Morphological data

Morphological data describe cellular and multicellular structure using quantitative measurements such as area, perimeter, circularity, texture, colony shape, and intercellular spacing. Cell and aggregate morphology reflects cellular state, developmental progression, culture heterogeneity, and product quality. Morphological information is typically acquired from images or videos and represented as quantitative features for classical ML models. Alternatively, deep learning models may learn representations directly from raw images or videos.

#### Biochemical and functional data

Biochemical and functional data characterize cellular composition and physiological activity, providing complementary information about differentiation, maturation, and disease. Biochemical data include measurements of proteins, metabolites, cytokines, nutrients, and other biochemical constituents. Functional data include electrophysiological recordings, calcium-transient measurements, contractility measurements, impedance-based measurements, biomagnetic recordings, and other indicators of cellular physiological activity. Depending on the acquisition modality, these data are typically represented as structured tabular data or sequential recordings suitable for ML analysis.

#### Experimental process metadata

Experimental process metadata describe the experimental design, culture conditions, and process parameters associated with SC experiments. Representative metadata include cell line, donor characteristics, passage number, treatment protocols, culture duration, media composition, environmental conditions, and bioprocess parameters. These variables provide essential context for biological interpretation, experimental reproducibility, and ML analysis and may themselves serve as predictive features for modeling SC behavior and differentiation outcomes. Experimental process metadata are typically represented as structured tabular data.

### SC data sources

SC research relies on diverse laboratory and clinical data sources that generate complementary biological and clinical information (Fig. 2). These sources include molecular and cellular profiling, biomedical imaging, biochemical profiling, functional measurements, and clinical resources, producing data represented as images, videos, structured tabular data, or sequential recordings for ML analysis. The principal data sources and representative acquisition technologies are summarized below.

**Fig. 2.**
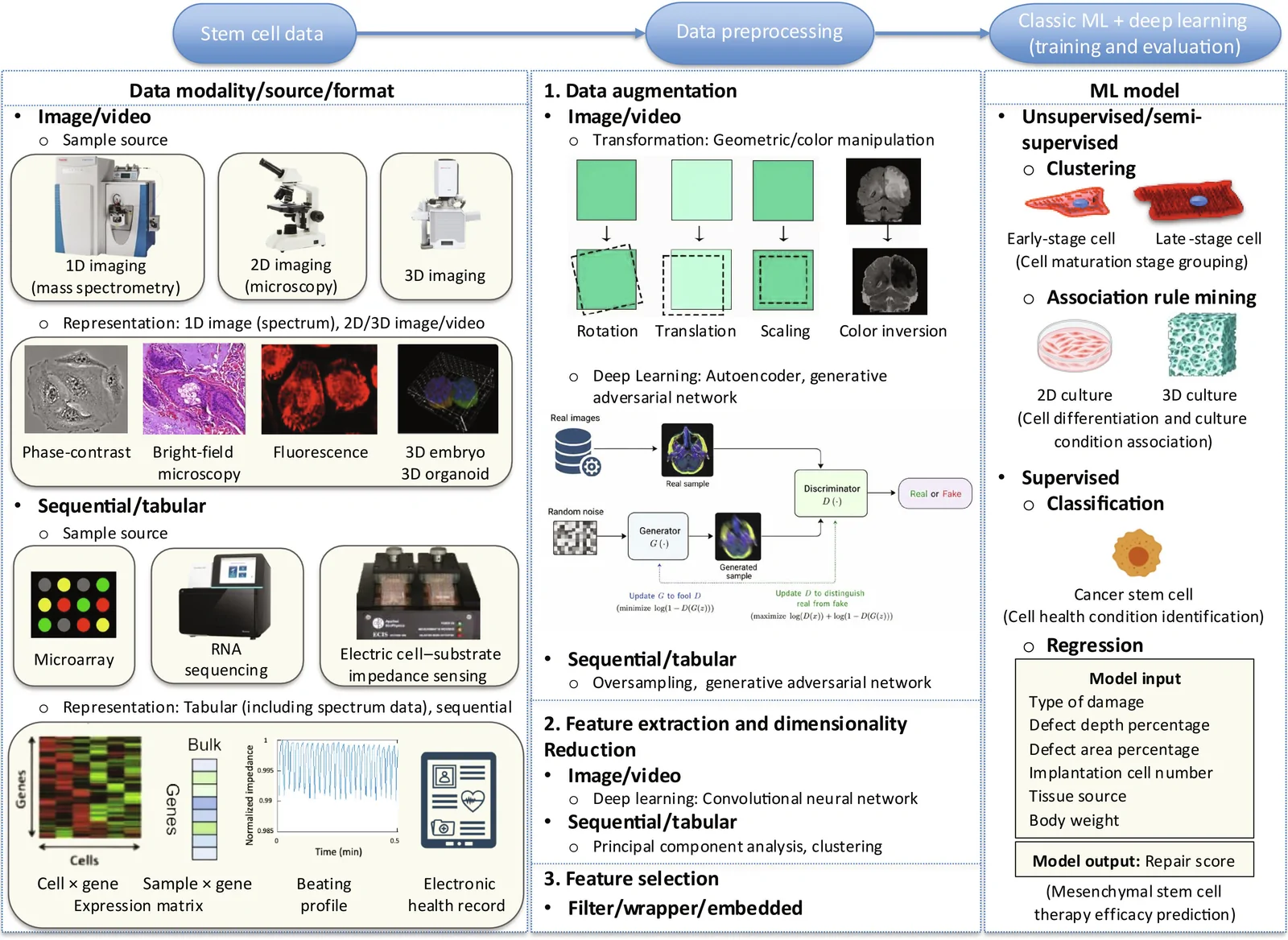
Machine learning (ML) workflow for stem cell (SC) research. SC data acquired from diverse experimental and clinical data sources are transformed into ML-ready data representations through preprocessing and feature engineering before model development using different learning paradigms and ML model families.

#### Molecular and cellular profiling sources

Molecular and cellular profiling sources generate transcriptomic, epigenomic, genomic, and cellular phenotypic data for characterizing SC identity, state, and function. Representative examples include sequencing technologies such as bulk RNA sequencing and single-cell RNA sequencing, molecular profiling technologies such as gene expression microarrays, DNA methylation profiling, and reverse transcription quantitative polymerase chain reaction, as well as cellular profiling techniques such as flow cytometry [15,16]. These technologies generate high-dimensional molecular or cellular measurements that are typically represented as structured tabular data for ML analysis, whereas imaging flow cytometry generates image-based data.

#### Biomedical imaging sources

Biomedical imaging sources generate morphological information for assessing SC morphology, differentiation, maturation, organoid development, culture quality, and therapeutic outcomes [17]. Typical laboratory imaging modalities include bright-field, phase-contrast, fluorescence, and confocal microscopy, whereas translational and clinical studies additionally employ magnetic resonance imaging, computed tomography, and positron emission tomography. These imaging technologies generate 2- or 3-dimensional images and videos, from which quantitative image features may subsequently be extracted for ML analysis.

#### Biochemical profiling sources

Biochemical profiling sources quantify proteins, metabolites, and other biochemical constituents to characterize cellular metabolism, signaling, and functional state. Typical acquisition methods include proteomic and metabolomic platforms, Raman spectroscopy, nuclear magnetic resonance spectroscopy, gas chromatography–mass spectrometry, and other targeted laboratory assays [18]. These measurements are typically organized as structured tabular inputs for downstream ML models. Spectroscopic measurements (i.e., spectra) may alternatively be represented as one-dimensional signals or transformed into image-like representations for deep learning models.

#### Functional measurement sources

Functional measurement sources characterize cellular physiological activity, including electrophysiological, mechanical, calcium-signaling, impedance-based, and biomagnetic measurements. Common examples include electrophysiological recordings, calcium imaging and calcium-transient recordings, contractility assays, impedance-based techniques such as electric cell–substrate impedance sensing, and biomagnetic measurements [19]. Raw outputs are commonly acquired as sequential recordings and are typically processed into structured tabular data suitable for ML analysis.

#### Clinical data sources

Clinical data sources used in SC research include electronic health records, clinical trial datasets, clinical registries, and other structured healthcare repositories. These resources provide patient-level information that complements SC-derived biological data and enables translational ML analyses linking experimental findings with patient outcomes and clinical decision support. Clinical data typically include patient demographics, diagnoses, laboratory measurements, medications, treatment histories, donor characteristics, transplantation variables, and clinical outcomes. Unlike SC-derived data, which characterize intrinsic cellular properties, clinical data describe patient characteristics, clinical interventions, and healthcare outcomes. For ML applications, they are typically represented as structured tabular data or longitudinal sequences.

## Statistical and ML Models in SC Research

SC datasets are heterogeneous, high-dimensional, and increasingly multimodal, requiring tailored computational methodologies. Consequently, model development depends on the SC data type, experimental data source, data representation, sample size, preprocessing strategy, feature representation, class balance, and validation protocol. Rather than identifying a universally superior algorithm, this section summarizes the computational methodologies commonly used in SC research, including data preprocessing, feature engineering, learning paradigms, ML model families, model selection, and evaluation strategies.

### Data preprocessing and augmentation

Data preprocessing transforms raw experimental measurements into ML-ready data representations. Because preprocessing requirements depend primarily on the data representation (images, videos, tabular data, sequential recordings, or multimodal representations), different SC data types and experimental data sources require distinct preprocessing pipelines. Appropriate preprocessing improves data quality, reduces technical variability, preserves biologically meaningful information, and enhances model robustness and reproducibility.

#### Image and video preprocessing

Image and video data acquired from biomedical imaging sources commonly require intensity normalization, denoising, resizing, background correction, segmentation, and region-of-interest extraction. These steps reduce technical variability arising from cell density, colony morphology, imaging conditions, and culture protocols while preserving biologically meaningful phenotypic information associated with pluripotency, differentiation, and maturation. Data augmentation methods, including flipping, rotation, cropping, translation, scaling, and brightness adjustment, expand training datasets and improve model robustness and generalization when biological characteristics are preserved [20,21]. Segmentation identifies biological structures of interest, whereas augmentation generates additional training samples through controlled image transformations.

#### Tabular data preprocessing

Tabular data commonly require quality filtering, normalization, transformation, scaling, imputation, feature filtering, and dimensionality reduction before ML analysis [22,23]. The specific preprocessing pipeline depends on the underlying experimental data source and biological measurements. For example, transcriptomic and epigenomic datasets typically require platform-specific normalization and batch-effect correction, whereas functional measurements often require extraction of physiologically meaningful descriptors before normalization. Experimental process metadata and clinical data generally involve categorical encoding, imputation, and feature standardization. In single-cell studies, dimensionality-reduction methods facilitate visualization and downstream analysis while preserving biologically meaningful cellular heterogeneity.

#### Sequential data preprocessing

Sequential recordings generated from electrophysiological, calcium-imaging, biomagnetic, impedance-based, and contractility measurements commonly undergo filtering, normalization, temporal alignment, frequency-domain analysis, and feature extraction before ML analysis [19]. Raw recordings preserve temporal relationships within the data, whereas processed measurements are often summarized as structured tabular features for subsequent ML analysis.

#### Multimodal data preprocessing

Multimodal preprocessing integrates complementary SC data from multiple experimental data sources and data representations. Preprocessing therefore combines modality-specific preprocessing with sample matching, temporal alignment, feature scaling, missing-modality handling, and multimodal fusion. Early, intermediate, and late fusion strategies integrate complementary information before, during, or after model training, respectively, thereby enabling effective integration of heterogeneous SC datasets while leveraging the complementary strengths of each modality [24].

### Feature engineering

Feature engineering transforms preprocessed data into informative feature representations for ML analysis. Depending on the data representation, this process may involve feature selection, feature extraction, or dimensionality reduction to improve predictive performance, reduce redundancy, and enhance biological interpretability. For multimodal data, feature engineering may be performed independently within each modality before multimodal feature fusion.

#### Feature selection

Feature selection reduces redundancy, improves computational efficiency, and enhances model interpretability by identifying the most informative features for downstream ML analysis. Common approaches include filter, wrapper, and embedded methods [25]. Filter methods rank features independently of the final ML model using statistical criteria such as correlation analysis, mutual information, chi-square tests, analysis of variance, and variance-based filtering. Their computational efficiency makes them particularly suitable for high-dimensional transcriptomic and other tabular datasets, although they generally do not capture interactions among features.

Wrapper methods instead evaluate candidate feature subsets through repeated model training and validation. Widely used approaches include recursive feature elimination and Monte Carlo feature selection. Although computationally more demanding, they account for feature interactions and often provide improved predictive performance. Hybrid approaches combining filter and wrapper methods can further improve computational efficiency.

Embedded methods perform feature selection during model optimization. Representative examples include least absolute shrinkage and selection operator, Elastic-Net, other regularized linear models, and tree-based feature importance measures. Because feature selection is incorporated directly into model training, embedded methods provide an effective balance between computational cost, predictive performance, and biological interpretability.

#### Feature extraction and dimensionality reduction

Unlike feature selection, which identifies informative variables, feature extraction transforms preprocessed measurements into compact feature representations while preserving informative biological variation. For image and video data, feature extraction may involve handcrafted morphological descriptors or learned convolutional neural network (CNN) representations [26]. For high-dimensional tabular data, principal component analysis and related methods reduce redundancy and facilitate visualization, whereas autoencoders learn nonlinear latent representations capable of capturing more complex biological relationships. These approaches improve computational efficiency, reduce redundancy, and preserve informative biological variation for subsequent ML analysis.

### Learning paradigms

Learning paradigms determine how ML models utilize available SC data during training and are broadly categorized into supervised, semisupervised, and unsupervised learning. The appropriate paradigm depends primarily on label availability, prediction task, and dataset characteristics.

#### Supervised learning

Supervised learning uses labeled data to learn predictive relationships between input features and known outcomes by solving classification or regression tasks. Classification predicts categorical outcomes such as cell type, differentiation stage, or disease status, whereas regression estimates continuous outcomes including differentiation efficiency, metabolite concentration, and treatment response. Because many SC studies include experimentally determined labels or quantitative measurements, supervised learning remains the most widely adopted learning paradigm.

#### Semisupervised learning

Semisupervised learning combines limited labeled data with larger unlabeled datasets to improve predictive performance [27]. This paradigm is particularly valuable for SC research because annotation of microscopy images and single-cell datasets is often labor-intensive. By incorporating unlabeled samples during training, semisupervised learning expands the effective training dataset and improves model generalization.

#### Unsupervised learning

Unsupervised learning identifies underlying structure in unlabeled data by discovering clusters or learning latent representations. Representative clustering methods, including *K*-means, Gaussian mixture models, hierarchical clustering, graph-based clustering, and density-based algorithms, identify cell states, developmental trajectories, and biologically meaningful subpopulations. Dimensionality-reduction and visualization methods such as principal component analysis, t-distributed stochastic neighbor embedding, and uniform manifold approximation and projection facilitate exploration and interpretation of high-dimensional SC data.

### ML model families

ML model families define the computational architectures used to implement different learning paradigms. They differ in representational capacity, computational requirements, interpretability, and data requirements. Representative model families commonly applied in SC research are summarized below.

#### Classical ML

Classical ML models remain widely used in SC research because they provide computational efficiency, robustness, and interpretability, particularly for high-dimensional tabular datasets with limited sample sizes. Classical ML encompasses linear, kernel-based, instance-based, tree-based, and ensemble methods.

##### Linear, kernel-based, and instance-based models

Representative approaches include regularized linear models, support vector machines, Gaussian process regression, and *k*-nearest neighbors. These models provide complementary strengths, including interpretable feature selection, nonlinear decision boundaries, predictive uncertainty estimation, and flexible instance-based learning [28–30].

##### Tree-based and ensemble models

Tree-based and ensemble methods include decision trees, random forests, extreme gradient boosting, extra trees, and related boosting approaches. These models effectively capture nonlinear feature interactions while providing robust predictive performance and interpretable feature importance measures, making them well suited to biomarker discovery, SC characterization, differentiation analysis, and clinical prediction tasks.

#### Deep learning

Deep learning models learn hierarchical feature representations directly from raw data and have become the dominant approach for analyzing complex biomedical images, videos, sequential recordings, and multimodal datasets.

##### Convolutional and recurrent neural networks

Common architectures include CNNs (e.g., AlexNet, VGG, ResNet, DenseNet, ResNeSt, and Xception) for image analysis; encoder–decoder networks (e.g., U-Net) for image segmentation; recurrent neural networks (RNNs), long short-term memory (LSTM) networks, and attention-based bidirectional LSTM for sequential modeling [20,31]. These architectures enable automated feature learning and end-to-end prediction from biomedical images, videos, and physiological recordings.

##### Generative, transformer-based, and foundation models

Advanced deep learning architectures include generative adversarial networks, conditional generative adversarial networks (e.g., pix2pix), variational autoencoders, vision transformers, and transformer-based foundation models such as Geneformer [5], scGPT [6], scFoundation [7], and BioMedGPT [32]. Foundation models leverage large-scale pretraining to learn transferable biological representations that can be adapted to diverse SC applications through fine-tuning. Collectively, these architectures support data augmentation, image synthesis and translation, representation learning, transfer learning, multimodal integration, and large-scale biological modeling across diverse SC datasets.

### Comparative model selection across data representations

Model selection is guided by the SC data representation, prediction task, dataset size, interpretability requirements, computational resources, and validation strategy. Representative model families for different SC data representations are summarized below.

#### Image and video representations

Biomedical images and videos are commonly analyzed using CNN-based architectures because convolution exploits local spatial structure through weight sharing and local receptive fields, providing strong inductive biases that make CNNs effective for many microscopy datasets, particularly when annotated training data are limited. Vision transformers become increasingly attractive as larger annotated imaging datasets or pretrained models become available because self-attention models long-range spatial relationships without relying on the locality assumptions imposed by convolution. Time-lapse imaging additionally benefits from CNN–RNN hybrid architectures that jointly model spatial and temporal cellular dynamics. When deep learning is not feasible because of limited sample sizes, computational constraints, or interpretability requirements, image and video data may be transformed into handcrafted feature representations prior to analysis. Images may be represented as tabular morphological or texture features, whereas videos may be represented as sequential descriptors or aggregated tabular features [33,34]. Although these transformations enable classical ML methods, they may discard informative spatial or temporal structure.

#### Tabular data

Structured tabular data derived from transcriptomic, epigenomic, biochemical, functional, experimental, and clinical measurements are commonly analyzed using classical ML methods because they provide robust predictive performance together with biological interpretability, particularly for moderate-sized datasets. Transformer-based and foundation models are increasingly applied to large-scale transcriptomic and other high-dimensional molecular datasets because self-attention effectively models dependencies among high-dimensional input features, while large-scale pretraining enables transferable biological representations for a wide range of SC applications [35]. In contrast, many clinical tabular datasets continue to rely primarily on classical ML methods because of their relatively limited sample sizes, structured feature representations, and interpretability requirements.

#### Sequential data

Sequential recordings generated from functional measurements benefit from architectures that directly model temporal dependencies. Classical ML remains effective when physiologically meaningful summary features are extracted, whereas RNN-, LSTM-, and transformer-based architectures learn temporal dependencies directly from sequential recordings. Transformer-based architectures are particularly well suited to long sequential recordings because self-attention captures long-range temporal dependencies while enabling parallel training, although they generally require greater computational resources than recurrent architectures.

#### Multimodal data

Multimodal datasets integrate complementary SC data from multiple experimental sources represented as images, videos, tabular data, and sequential recordings. Model selection therefore depends on modality correspondence, data completeness, computational resources, and the desired balance between predictive performance and interpretability. Multimodal fusion strategies remain widely used for integrating heterogeneous modalities, whereas recent multimodal transformer architectures increasingly enable end-to-end joint representation learning across molecular, morphological, biochemical, functional, and clinical data [36].

### Statistical analysis and model evaluation

Performance evaluation depends on the learning task, prediction objective, data representation, and validation strategy (Table 4). Accordingly, different evaluation metrics are used for classification, regression, unsupervised learning, and segmentation tasks. Classification models are primarily used to distinguish cell identities, developmental stages, and SC quality [37–41]; identify disease phenotypes [42,43]; and predict treatment responses [44,45]. Regression models predict continuous biological or clinical outcomes, including gene expression [46], cellular maturation [47], SC potency [48], disease progression [49], and treatment efficacy [50]. Unsupervised learning identifies cellular subpopulations, phenotypic heterogeneity, and developmental trajectories [51–53], whereas segmentation models delineate cells, colonies, or tissue structures from microscopy images [54,55]. Statistical analyses complement ML evaluation by assessing the significance, robustness, and reliability of observed biological differences.

**Table 4.** Common evaluation metrics used in machine-learning-based stem cell research

Category	Abbreviation	Evaluation metric: definition
Statistics	FDR	False discovery rate: Expected proportion of false positives among statistically significant findings
	*P*	*P* value: Probability of observing a result at least as extreme as the one obtained, assuming that the null hypothesis is true
Classification	Accuracy	Proportion of correctly classified samples among all evaluated samples
	Precision	Proportion of predicted positive samples that are true positives (positive predictive value)
	Recall	Sensitivity: Proportion of actual positive samples correctly identified (true positive rate)
	F1	F1-score: Harmonic mean of precision and recall, particularly useful for imbalanced datasets
	AP	Average precision: Summary metric of the precision–recall curve, computed as weighted mean of precision over increasing recall
	AUC	Area under the receiver operating characteristic (ROC) curve: Measures discrimination ability across all classification thresholds
	MCC	Matthews correlation coefficient: Measures the correlation between observed and predicted binary classifications
Regression	*R* ^2^	Coefficient of determination: Proportion of variance in the response variable explained by the regression model
	MAE	Mean absolute error: Average absolute difference between predicted and observed values
	RMSE	Root mean squared error: Square root of the average squared difference between predicted and observed values
	*r*	Pearson correlation coefficient: Measures the strength and direction of the linear relationship between 2 continuous variables
Unsupervised	SC	Silhouette coefficient: Measures cluster cohesion and separation based on intracluster and nearest-cluster distances
Segmentation	IoU	Intersection over union: Ratio of the overlap between predicted and ground-truth segmentation regions to their union

Validation strategies evaluate model generalizability across independent biological and experimental conditions. The appropriate validation strategy depends on the intended application and the availability of suitable independent datasets. Studies using publicly available molecular datasets (e.g., transcriptomic and epigenomic datasets) or multicenter clinical registries are generally better positioned to perform external validation, whereas microscopy-based studies and laboratory-specific functional assays often rely on carefully designed internal validation because independent datasets remain scarce. SC datasets are inherently influenced by donor variability, cell-line differences, culture conditions, and experimental protocols; consequently, validation across independent biological sources is generally more informative than evaluation based solely on randomly partitioned samples from a single dataset. Consequently, the absence of external validation does not necessarily diminish the scientific value of exploratory biological studies or methodological developments. However, for models intended to support clinical decision-making, treatment-response prediction, or deployment across laboratories or clinical settings, independent external validation is essential to demonstrate robust generalizability across donors, cell lines, experimental protocols, institutions, and patient populations.

## Research Domain I: SC Biology and Engineering

ML increasingly advances SC research by enabling quantitative analysis of SC data throughout the developmental process. Rather than reviewing individual algorithms or studies, with representative studies organized in Table 5, this section examines how ML contributes to 4 major stages of SC biology: reprogramming and pluripotency, differentiation and lineage specification, postdifferentiation maturation, and quality control.

**Table 5.** Summary of representative machine learning (ML) studies in Research Domain I: SC Biology and Engineering. Studies are organized first by primary data source and then by research objective. * denotes public availability; † denotes transformation between data representations.

Data source	Research objective	ML model/pipeline	Reported performance	Validation
Biological stage 1: Reprogramming and pluripotency				
Transcriptomic and epigenomic data: Tabular representation unless specified otherwise				
[38] Gene expression microarray*	mPSC and nonpluripotent mouse cells: Pluripotency classification and biomarker identification	Genetic algorithm/SVM + linear SVM	Accuracy: Up to 100%	CV
[51] Single-cell mass cytometry*	mESC: Pluripotent-state identification and cell-state characterization	PCA + GMM	-	US
[58] Gene expression microarray + ChIP-seq*	mESC: Self-renewal and pluripotency gene prediction	RBF-SVM	Accuracy: 94%	CV
[57] Epigenomic profiling + DNA sequence composition*	H1 hESC: Epigenetic regulation of gene expression	RBF-SVM	Accuracy: 95.80%; MCC: 0.920	IHO
[56] DNA methylation profiling*	hiPSC and hESC: Pluripotency assessment and epigenetic signature identification	AROW	Accuracy: 94.23%	CV
Morphological data: Image representation unless specified otherwise				
[63] Transmitted-light microscopy	mESC and miPSC: Pluripotent-state classification	ResNet50	Accuracy: *>*90%	IHO
[60] Time-lapse bright-field + fluorescence microscopy*^†^	MEF → miPSC: iPS-progenitor identification during reprogramming	XGBoost	AP: 0.523–0.750	IV
[61] Time-lapse bright-field microscopy (video)^†^	MEF → miPSC Human urine-derived cell → hiPSC: Colony formation detection and reprogramming-stage prediction	AlexNet + HMM	*r*: 0.877	IV
[59] Phase-contrast microscopy*^†^	hPSC colony: Colony phenotype classification	RF	Accuracy: 99% ± 2%; AUC: 0.997	CV
[46] Phase-contrast microscopy^†^	PBMC-derived hiPSC: Gene expression prediction and iPSC quality evaluation	VQ-VAE-2 + SVR	*R*^2^: 0.685	IHO
[62] Time-lapse phase-contrast microscopy (video)	hESC: Culture-condition and pluripotent cell-state classification	Autoencoder + RandNet	Accuracy: 96.28%	IHO
Biological stage 2: Differentiation and lineage specification				
Transcriptomic and epigenomic data: Tabular representation unless specified otherwise				
[67] DNA methylation profiling*	hiPSC: Differentiation-state assessment and lineage classification	SteMClass (RF)	Accuracy: 76.5%	EV
[69] Gene expression microarray*	NSC: Molecular-signature identification and lineage classification	MCFS + SVM	MCC: 0.918	CV
[66] scRNA-seq*	mHSC: HSC identification and annotation	hscScore (MLP)	–	EV
[70] scRNA-seq and CITE-seq*	hPSC-derived HSPC: HSPC and HSC-like cell identification and annotation	ANN	–	EV
[64] scRNA-seq*	hiPSC → cardiomyocyte: Differentiation-stage prediction	Elastic-Net sparse regression	Accuracy: 0.840–0.940	IHO
[71] Paired daughter-cell gene expression profiling*	Mouse long-term hematopoietic stem cell: HSC division-pattern classification and lineage-fate analysis	ANN	Accuracy: 89%	CV
[37] RNA-seq*	hMSC → multiple lineages: Lineage-fate prediction	MeD-P (KNN)	Accuracy: 90.63%	EV
[68] Longitudinal single-cell RT-qPCR profiling*	mESC → epiblast-like cell → NPC: Differentiation-dynamics modeling	HMM	–	US
Morphological data: Image representation unless specified otherwise				
[72] Transmitted-light microscopy	mESC → epiblast-like cell: Very early differentiation detection	Augmentation + ResNet50	Accuracy: 99.6%	IV
[34] Dual-fluorescence time-lapse microscopy (video)^†^	MuSC: Cell-fate transition tracking and division classification	Dual-FLIT (*K*-means + 1-NN)	–	NR
[75] Phase-contrast microscopy^†^	hiPSC and hiPSC-derived cells: Cell-lineage classification and RPE differentiation assessment	CNN + PCA + SVM	Accuracy: 97.8%	IHO
[77] Differential-interference-contrast microscopy	Mouse LT-HSC, ST-HSC, and MPP: Hematopoietic subpopulation classification	LSM (ResNet50)	F1: 0.740	IHO
[78] Bright-field microscopy	hMSC → adipogenic and osteogenic lineages: Lineage prediction	ResNet50	Accuracy: 95.7%	IHO
[74] Single-cell confocal fluorescence microscopy	hNTSC: Multipotency classification and donor comparison	DenseNet121	Accuracy: 85.98%	IHO
[76] Bright-field imaging flow cytometry*	Rat NSC: Differentiation-fate prediction	Xception	Accuracy: 92.3%	IHO
[79] Bright-field microscopy	hiPSC → kidney organoid: Kidney organoid differentiation-status prediction	DenseNet121	*r*: 0.783	CV
[73] Phase-contrast microscopy	hPSC → hepatocyte: Hepatocyte differentiation-stage classification	Custom CNN	Accuracy: 95%; F1: 0.950	IHO
Biochemical and functional data: Tabular representation unless specified otherwise				
[80] GC–MS metabolomics	Human umbilical cord blood MSC → osteogenic lineage: Osteogenic differentiation-status prediction	XGBoost	AUC: 0.826	EV
[83] Electrical impedance spectroscopy (sequential)	hiPSC-derived neural precursor cell differentiation- versus proliferation-state classification	LSTM	Accuracy: Up to 97%	IV
Experimental process metadata: Tabular representation unless specified otherwise				
[82] Published experimental metadata*	Mammalian MSC: Differentiation-fate prediction	CARM	Accuracy: 77.04%	CV
[81] Donor and culture-process metadata	hMSC: Population-doubling-time prediction	RF	MAE: 0.700–0.870 d	IHO
[84] Bioprocess measurements and experimental process metadata	hPSC → cardiomyocyte: Cardiomyocyte-content prediction	RF	Accuracy: 89%; precision: 92%	IHO
Biological stage 3: Postdifferentiation maturation				
Transcriptomic and epigenomic data: Tabular representation unless specified otherwise				
[47] miRNA expression profiling*	hiPSC-CM: Postdifferentiation maturation assessment	XGBoost	RMSE: 4.461 d; *R*^2^: 0.972	IHO
[85] scRNA-seq*	MuSC: Cell age classification and activation dynamics analysis	L1-regularized SVM	Accuracy: ∼95%	IHO
Morphological data: Image representation unless specified otherwise				
[39] Bright-field microscopy (video)^†^	hiPSC-CM: Maturation-stage classification	GAN + CNN	Accuracy: 92.9%	IV
[52] Confocal fluorescence microscopy^†^	hiPSC-CM: Maturation phenotype clustering	*K*-means, GMM	SC: 0.170	US
[86] Wound-healing assay microscopy (video)^†^	Human and nonhuman primate NPCs: Cross-species migration analysis	VGG19 + cell tracking	–	NR
Biochemical and functional data: Sequential representation unless specified otherwise				
[87] SQUID biomagnetic recordings + numerical simulation	miPSC-CM: Weak biomagnetic-signal peak detection	LSTM	AUC: 0.961	IV
[88] Optical mapping	hESC-CM: Atrial-like versus ventricular-like phenotype classification	Semi-LSTM-M	Agreement (*vs*. reference classifier): 94.73%	CV
[89] Single-cell Raman microspectroscopy (tabular)*	hiPSC, hiPSC-derived NSC, and neuron: Neural developmental-stage classification	t-SNE + stacked SGB	Accuracy: 97.5%	IHO
Biological stage 4: Quality control				
Transcriptomic and epigenomic data: Tabular representation unless specified otherwise				
[90] RT-qPCR gene expression profiling*	hiPSC and trilineage derivatives: Pluripotency and differentiation-state quality assessment	hiPSCore	Accuracy: up to 100%	IV
Morphological data: Image representation unless specified otherwise				
[40] Phase-contrast microscopy^†^	hBMSC: Culture-quality phenotype classification	RBF-SVM + LR/LDA + KNN	AUC: 0.816 (day 2); AUC: 0.787 (day 4)	IHO
[95] Phase-contrast microscopy^†^	hiPSC: Colony-quality grading	SIFT + KNN	Accuracy: 62.4%	CV
[54] Phase-contrast microscopy^†^	hiPSC: Culture-status segmentation	U-Net	IoU: 0.777; F1: 0.753	IHO
[94] Bright-field microscopy	hiPSC-CM: Culture-quality classification	VGG16	F1: 0.890 ± 0.010	CV
[93] Live-cell bright-field microscopy	hNTSC: MUSE-marker-based functional screening	DenseNet121 + Bayesian optimization	AUC: 0.975; F1: 0.922	IHO
[91] Bright-field contractility microscopy (video)^†^	hiPSC-CM: Contractile-function quality classification	FFT + UMAP + RBF-SVM	Accuracy: 0*.*89 ± 0*.*02; F1: 0*.*82 ± 0*.*04	CV
[92] Longitudinal bright-field microscopy (video)*^†^	hPSC-derived CPC and cardiomyocyte: Differentiation quality monitoring and lineage recognition	pix2pix (cGAN); ResNeSt101 + Grad-CAM	Accuracy (CPC): 71*.*9% ± 12*.*0%; *r* (cardiomyocyte): 0.930	IHO
[53] Confocal fluorescence microscopy^†^	hBMSC: Microenvironment-associated cell-shape classification	Supercell + SVM	Accuracy: 97% ± 2%	CV
[96] 3D high-content fluorescence microscopy^†^	mPSC-derived embryo-like structure: Structure-quality classification	PhenoLOGIC	Accuracy: 92%; F1: 0.800	IHO
[41] Imaging flow cytometry	SHED and human Wharton’s jelly MSC: Cell-source classification and morphometric-heterogeneity assessment	CNN	Accuracy: 97.54%; F1: 0.9698	CV
Biochemical and functional data: Tabular representation unless specified otherwise				
[97] Multiparameter flow cytometry	Cord-blood CD34^+^ HSPC: Automated CD34^+^ cell enumeration	AGA	*r*: 0.991	IV
[48] NMR spectroscopy + mass spectrometry metabolomics*	hMSC: Immunomodulatory-potency prediction and metabolite biomarker identification	Consensus regression	*R*^2^: up to 1.000	CV

Validation abbreviations: External/independent: EV, external validation; IV, independent validation using an independent dataset. Internal: IHO, internal holdout evaluation (including train–test splits, held-out test sets, and repeated random subsampling/Monte Carlo validation); CV, cross-validation (including nested, leave-one-out, and repeated *k*-fold variants). Other: US, unsupervised evaluation; SIM, simulation-based evaluation; NR, validation not reported. ChIP-seq, chromatin immunoprecipitation followed by sequencing; scRNA-seq, single-cell RNA sequencing; CITE-seq, cellular indexing of transcriptomes and epitopes by sequencing; RT-qPCR, reverse transcription quantitative polymerase chain reaction; GC–MS, gas chromatography–mass spectrometry; miRNA, microRNA; SQUID, superconducting quantum interference device; NMR, nuclear magnetic resonance

### Reprogramming and pluripotency

Reprogramming converts differentiated somatic cells into PSCs capable of unlimited self-renewal and multilineage differentiation. This process is inherently inefficient, asynchronous, and characterized by heterogeneous intermediate cell states, making reliable identification of successfully reprogrammed cells a major challenge. Conventional evaluation relies on gene expression and epigenetic profiling, immunostaining, reporter systems, colony morphology, and expert interpretation. Computational approaches have traditionally employed statistical classifiers and handcrafted molecular or morphological features for specific reprogramming tasks, whereas recent ML approaches increasingly leverage high-dimensional molecular profiling and deep-learning-based image analysis. Although these methods improve quantitative assessment, they generally depend on destructive endpoint assays, manually engineered features, or protocol-specific analytical frameworks that limit continuous and scalable monitoring.

The reviewed ML applications during reprogramming are primarily based on 2 complementary data categories. Transcriptomic and epigenomic studies leverage gene expression profiling, epigenetic, single-cell molecular, and integrated regulatory data to characterize pluripotent identity, identify molecular determinants of pluripotency, and assess cell-state heterogeneity [38,51,56–58]. Morphology-based studies use label-free microscopy to identify reprogrammed cells, monitor colony formation and phenotype, predict gene expression patterns, and enable continuous, nondestructive assessment of reprogramming dynamics [46,59–63]. Together, these studies demonstrate that molecular profiling provides high-dimensional characterization of pluripotent identity, whereas morphology-based analysis enables continuous, label-free monitoring throughout the reprogramming process.

Overall, these findings demonstrate that ML complements conventional molecular assays and image analysis by combining quantitative molecular profiling with automated morphological phenotyping, thereby improving the accuracy, efficiency, and reproducibility of pluripotency assessment. Future advances are likely to require integrating molecular, morphological, and temporal data to enable earlier prediction of successful reprogramming, continuous tracking of cell-state transitions, and more robust assessment of pluripotency.

### Differentiation and lineage specification

Differentiation commits SCs toward specialized lineages through coordinated molecular, structural, and functional changes regulated by dynamic gene expression programs and interactions with the cellular microenvironment. Differentiation involves a broader range of biological processes, including lineage commitment, cell-state transitions, functional maturation, and manufacturing optimization. Therefore, computational approaches extend beyond molecular assays and microscopy to include statistical learning, trajectory inference, and gene-regulatory-network models for describing lineage progression and cell-fate dynamics [64,65]. Although these approaches have provided valuable biological insights, they often rely on predefined mathematical assumptions or handcrafted analytical frameworks that may not generalize across heterogeneous differentiation systems.

Reflecting this broader biological scope, ML has been applied across 4 major SC data types. Transcriptomic, epigenomic, and single-cell molecular profiling studies characterize differentiation states, identify lineage-associated molecular signatures, annotate hematopoietic stem and progenitor cell populations, and model lineage commitment and differentiation trajectories [37,64,66–71]. Morphology-based studies use label-free and fluorescence microscopy to monitor differentiation progression, classify lineage identity, predict differentiation outcomes, and assess organoid development directly from cellular morphology [34,72–79]. Biochemical and functional studies evaluate lineage commitment through metabolomic and physiological measurements, whereas experimental process metadata support prediction of differentiation outcomes, cell expansion kinetics, and manufacturing performance [80–84].

Collectively, these studies illustrate the versatility of ML across molecular, morphological, biochemical, functional, and process-related data. Compared with reprogramming, differentiation involves a wider spectrum of prediction tasks and data modalities, reflecting the increasing biological complexity of lineage specification and downstream manufacturing. The reviewed applications span diverse SC systems, including hematopoietic, neural, mesenchymal, cardiac, hepatic, retinal, and renal lineages, highlighting the broad applicability of ML throughout SC differentiation. Future advances are likely to depend on standardized differentiation protocols, multimodal characterization, and robust predictive models that generalize across diverse SC types and manufacturing workflows.

### Postdifferentiation maturation

Following differentiation and lineage specification, SC-derived cells must acquire adultlike molecular, structural, and functional properties before they can reliably model native tissues or support downstream applications such as disease modeling, drug screening, and regenerative medicine. Conventional maturation assessment relies on molecular profiling, structural imaging, electrophysiological measurements, metabolic assays, and functional testing. Although informative, these approaches often depend on destructive assays, endpoint measurements, or individual modalities that capture only selected aspects of cellular maturity.

As summarized in Table 5, ML-based maturation assessment spans 3 major SC data types. Transcriptomic and epigenomic studies use microRNA expression and single-cell molecular profiling to characterize maturation status and age-related cellular states [47,85]. Morphology-based studies apply microscopy to classify maturation stages, characterize phenotypic variation, and quantify migration behavior through automated image analysis [39,52,86]. Biochemical and functional studies use biomagnetic recordings, optical electrophysiological mapping, and Raman microspectroscopy to evaluate electrophysiological function and developmental maturity [87–89].

Together, these studies demonstrate that maturation is best characterized by integrating complementary molecular, morphological, and functional data rather than relying on a single modality. Unlike differentiation, which emphasizes lineage commitment and cell identity, maturation focuses on the progressive acquisition of lineage-specific structure and function. The reviewed applications span cardiac, neural, and skeletal muscle systems, indicating that ML-based maturation assessment is broadly applicable across diverse developmental systems. Continued development will rely on standardized maturity benchmarks, longitudinal assessment of developmental progression, and integration of complementary molecular, morphological, and functional measurements.

### Quality control

Quality control ensures that SC-derived products satisfy predefined standards of identity, purity, potency, consistency, and safety before experimental, industrial, or clinical use. Reliable quality assessment is essential for reproducible manufacturing, large-scale production, and regulatory compliance. Conventional quality-control workflows combine molecular assays, microscopy, flow cytometry, functional testing, and expert evaluation, but these approaches are often labor-intensive, destructive, or difficult to standardize across manufacturing pipelines.

ML-based quality control integrates 3 major SC data types to automate product evaluation. Transcriptomic and epigenomic studies use gene expression profiling to verify pluripotency and differentiation status [90]. Morphology-based studies represent the largest group of applications, enabling automated assessment of culture quality, colony morphology, differentiation status, functional phenotypes, and structural integrity through deep learning and feature-based image analysis [40,41,53,54,91–96]. Biochemical and functional studies complement image-based assessment by applying flow cytometry and metabolomic profiling to quantify cell populations, predict immunomodulatory potency, and identify biomarkers associated with critical quality attributes of SC products [48,97].

Collectively, these applications illustrate how ML is evolving from a tool for biological characterization into an integral component of SC manufacturing and quality assurance. The reviewed applications encompass PSCs together with multiple differentiated cell products, highlighting the broad applicability of ML for standardized quality assessment across diverse manufacturing workflows. Continued advances are expected to enable real-time quality monitoring, anomaly detection, and closed-loop manufacturing systems that support scalable, reproducible, and high-quality SC production.

Together, these 4 biological stages illustrate the progressive integration of ML across the SC developmental continuum, from reprogramming and lineage specification to maturation and quality control. As biological complexity increases across these stages, so does the diversity of SC data types, analytical objectives, and validation strategies. Future progress will depend on multimodal data integration, standardized experimental protocols, interoperable data resources, and rigorous external validation to enable scalable and clinically translatable SC research.

## Research Domain II: Clinical Applications

ML further extends SC research from SC biology and engineering to clinical applications. As summarized in Table 6, disease modeling, therapeutic development, and clinical decision support represent successive stages of the translational continuum, progressing from mechanistic understanding to therapeutic evaluation and ultimately individualized patient management. Along this continuum, the analytical focus shifts from extracting biological patterns in controlled experimental systems to integrating heterogeneous real-world data, thereby placing greater emphasis on data quality, model generalizability, and rigorous validation.

**Table 6.** Summary of representative machine learning (ML) studies in Research Domain II: Clinical Applications. Studies are organized first by primary data source and then by research objective. * denotes public availability; † denotes transformation between data representations. Table conventions and validation abbreviations follow Table 5.

Data source	Research objective	ML model/pipeline	Reported performance	Validation
Application area 1: Disease modeling				
Transcriptomic and epigenomic data: Tabular representation unless specified otherwise				
[43] Gene expression microarray*	LSC: Disease classification	MCFS + SVM	Accuracy: 92.1%; MCC: 0.835	CV
[99] scRNA-seq + RNA-seq*	Lung CSC: CSC identification and prognosis prediction	LASSO–Cox	AUC: 0.680–0.740	IV
[101] scRNA-seq + gene expression microarray*	MSC-treated intervertebral disc degeneration: Disease-associated hub-gene identification	LASSO + RFE–SVM	Hub gene: LAPTM5	NR
[100] scRNA-seq + RNA-seq*	Prostate CSC: Stemness characterization and prognostic stratification	RF + NB	AUC: 0.660–0.880	EV
[102] Gene expression microarray + DNA methylation profiling*	Medulloblastoma CSC: Stemness characterization and prognostic modeling	OCLR + LASSO–Cox	AUC: 0.750–0.860	IHO
Morphological data: Image representation unless specified otherwise				
[42] Bright-field microscopy (video)^†^	Healthy and Timothy syndrome hiPSC-CM: Disease classification	DT	Accuracy: 92%; AUC: 0.960	CV
[103] Fluorescence microscopy	ALS and control hiPSC-derived motor neuron: Disease classification	VGG16	Accuracy: 90%; AUC: 0.970	IHO
[55] Phase-contrast + fluorescence microscopy	iPSC-derived CSC: CSC region segmentation	pix2pix (cGAN)	Precision: 0.550	NR
Biochemical and functional data: Sequential representation unless specified otherwise				
[106] Calcium imaging (image)^†^	Healthy and diseased hiPSC-CM: Functional phenotype analysis	Analytical algorithm + SVM	Accuracy: 87%; AUC: 0.950	IHO
[104] Calcium-transient recordings^†^	Healthy and diseased hiPSC-CM: Disease classification	RF	Accuracy: 87.4%	CV
[105] Calcium-transient recordings^†^	Inherited cardiac disease hiPSC-CM: Disease classification	RF	Accuracy: 87.6%	CV
[49] Flow cytometry (tabular)*	Colon and breast CSC: CSC-subpopulation development prediction	Genetic algorithm regressor	*R*^2^: 0.930–1.000	IHO
Application area 2: Therapeutic development				
Transcriptomic and epigenomic data: Tabular representation unless specified otherwise				
[45] RNA-seq + scRNA-seq*	CSC across tumor types: CSC abundance and potency profiling	OSCORP (SVR + SVM)	Accuracy: 98.67%; F1: 0.980	CV
[107] Gene expression perturbation profiling*	Breast CSC-like cell: Drug repurposing for differentiation induction	DREDDA	5/6 compounds	IV
[108] Gene expression profiling*	Murine HSPC: Viral-vector genotoxicity-risk prediction	SAGA (SVM)	Accuracy: 90.9%; AUC: 0.940	IV
Morphological data: Image representation unless specified otherwise				
[111] High-content fluorescence microscopy*	hiPSC-CM: Drug-induced cardiotoxicity screening	ResNet50	Accuracy: 97.0%	IHO
[112] Magnetic particle imaging	Transplanted hiPSC-derived islet organoid: *In vivo* graft tracking and quantification	Segmentation + *K*-means++	*R*^2^: 0.990	IV
[115] High-content fluorescence microscopy^†^	hiPSC-derived renal proximal tubular-like cell: Nephrotoxicity prediction	RF	Accuracy: 87%	CV
Biochemical and functional data: Sequential representation unless specified otherwise				
[109] Muscular thin-film contractility recordings^†^	hPSC-derived cardiac tissue: Cardioactive compound and mechanism classification	Binary + multiclass SVM	Accuracy: 71.7%	IHO
[44] Calcium-transient recordings*^†^	hiPSC-CM: Compound-induced cardiac-activity prediction	RF	Accuracy: 81%; AUC: 0.860; F1: 0.860	CV
[110] Calcium-transient recordings^†^	hiPSC-CM: Inotropic drug-response classification	Modified RF	Accuracy: 84%; AUC: 0.840	CV
[113] Voltage-sensitive-dye recordings*^†^	hiPSC-CM: Chronotropic-drug classification	TreeBagger	Accuracy: 75%	IHO
[114] Calcium-transient recordings^†^	Inherited cardiac disease hiPSC-CM: Dantrolene-response classification	RF	Accuracy: 79%	CV
[130] Simulated action-potential waveforms	iPSC-CM and adult-CM: Action-potential translation and drug-effect classification	LSTM-DNN	Accuracy: 92%	SIM
Clinical studies and evidence synthesis: Tabular representation unless specified otherwise				
[117] Preclinical studies*	MSC therapy for sepsis: Treatment-response rule discovery	J48 (DT)	Accuracy: 74.5%	CV
[50] Preclinical and clinical studies*	MSC therapy: Cartilage-repair efficacy prediction	ANN	*R*^2^: 0.637	CV
[116] Clinical trials*	Stem cell transplantation for spinal cord injury: Comparative efficacy and safety assessment	BNMA	Effects/treatment rankings	NR
[118] Published clinical studies*	Stem cell/scaffold therapy for spinal cord injury: Comparative efficacy assessment	BNMA	Effects/treatment rankings	NR
[119] Clinical trials*	MSC therapy for osteoarthritis: Comparative efficacy and safety assessment	BNMA	Effects/treatment rankings	NR
Application area 3: Clinical decision support				
Transcriptomic and epigenomic data: Tabular representation unless specified otherwise				
[120] Gene expression profiling*	HSPC transplantation: Transplantation-associated transcriptional signature identification	l_1_l_2FS_ (Elastic-Net)	Accuracy: 88%	CV
Morphological data: Image representation unless specified otherwise				
[33] Bone-marrow aspirate microscopy^†^	Acute myeloid leukemia (AML) after HSCT: Posttransplant relapse-risk prediction	RF + LDA	AUC: 0.800	CV
Biochemical and functional data: Tabular representation unless specified otherwise				
[121] Plasma protein expression profiling*	Severe COVID-19 patients receiving MSC therapy: Treatment-response prediction	LASSO + LR	AUC: 0.767	IHO
Clinical data: Tabular representation unless specified otherwise				
[122] Donor registry	Unrelated HSC donors: Donor availability prediction	BDT	AUC: 0.826	IV
[123] Donor records	Related allogeneic HSC donors: G-CSF-induced hematopoietic stem-cell mobilization prediction	RF	AUC: 0.890	CV
[124] HSCT registry	Allogeneic HCT recipients: 100-d mortality prediction	ADT	AUC: 0.700	IV
[125] Pretransplant records	AML patients undergoing allogeneic HSCT: 1-year relapse prediction	ADT	Accuracy: 71.0%; AUC: 0.667	IV
[31] Longitudinal clinical records (sequential)	AML: Short-term mortality and relapse prediction	Att-BLSTM + SVM	AUC: 0.770	CV
[126] Therapeutic drug-monitoring records	Pediatric HSCT recipients: Initial cyclosporine therapeutic-range prediction	TAN	AUC: 0.890	CV
[127] Pediatric transplant records*	Pediatric HCT recipients: Posttransplant survival prediction	Stacked ensemble	AUC: 0.920; F1: 0.880	IHO
[128] Transplant records	Adult allogeneic HSCT recipients: Long-term survival prediction	DERGA + extra trees	Accuracy: 93.26%	IHO
Multimodal data (clinical + genomic): Tabular representation unless specified otherwise				
[129] Clinical variables + SNP genotyping	HCT recipients: Posttransplant cognitive-impairment prediction	Elastic-Net + LR	AUC: 0.710	EV

ALS, amyotrophic lateral sclerosis; HSCT, hematopoietic stem cell transplantation; G-CSF, granulocyte-colony-stimulating-factor; HCT, hematopoietic cell transplantation; SNP, single-nucleotide polymorphism

### Disease modeling

Disease modeling provides the biological foundation for translational SC research by reproducing patient-specific pathological phenotypes under controlled *in vitro* conditions. Before the widespread adoption of ML, disease phenotyping primarily relied on manual microscopy, molecular assays, and high-content screening based on predefined image features and rule-based analyses [98]. Although these approaches have substantially advanced SC biology, they remain limited in their ability to capture cellular heterogeneity and integrate high-dimensional imaging, molecular, and functional data. ML enables more comprehensive, objective, and reproducible characterization of disease-associated phenotypes by automatically learning informative patterns from heterogeneous data.

Disease-modeling applications span 3 major SC data types. Transcriptomic and epigenomic studies leverage gene expression, single-cell molecular, and integrated molecular profiling to classify disease states, identify disease-associated molecular signatures and hub genes, characterize stemness in cancer stem cells (CSCs), and develop prognostic models [43,99–102]. Morphology-based studies use bright-field, fluorescence, and phase-contrast microscopy to classify disease phenotypes and segment CSC regions directly from cellular images [42,55,103]. Biochemical and functional studies employ calcium imaging, calcium-transient recordings, and flow cytometry to characterize functional phenotypes, predict disease progression, and identify CSC subpopulations [49,104–106].

Across these data modalities, the reviewed applications predominantly focus on cancer, particularly CSC and leukemic stem cell biology while also encompassing neurological, cardiac, musculoskeletal, and developmental disorders, including amyotrophic lateral sclerosis, Timothy syndrome, inherited cardiac diseases, and intervertebral disc degeneration. Taken together, these studies demonstrate the broad applicability of ML for quantitative disease phenotyping, biomarker discovery, functional characterization, and prognostic assessment across diverse SC-based disease models.

Predictive performance varied across studies according to the biological question, data modality, and analytical strategy. Imaging-based disease-classification studies generally achieved high discrimination, with areas under the curve (AUCs) of 0.950 to 0.970 and accuracies of 87% to 92% [42,103,106], likely reflecting the rich morphological and phenotypic information captured by microscopy. Transcriptomic prognostic models reported more moderate AUCs (0.660 to 0.880) [99,100,102], which may reflect the greater complexity of long-term outcome prediction. Functional analyses based on calcium-transient recordings achieved classification accuracies of approximately 87% to 88% [104,105], while gene-expression-based disease classification reached an accuracy of 92.1% with a Matthews correlation coefficient of 0.835 [43]. Regression analysis of CSC-subpopulation identification also demonstrated strong predictive performance (*R*^2^: 0.930 to 1.000) [49]. These performance variations primarily reflect differences in prediction tasks, biological complexity, data modalities, and validation strategies rather than inherent superiority of one ML approach over another.

Validation strategies largely reflected dataset availability and study design. Imaging-based studies primarily relied on cross-validation or internal holdout evaluation because independent image datasets remain limited [42,103,106], whereas transcriptomic studies more frequently incorporated external validation cohorts to assess model generalizability [99,100,102]. These observations highlight how dataset availability influences validation strategies while reinforcing the complementary value of molecular, morphological, biochemical, and functional data for comprehensive disease characterization. Together, they provide biologically interpretable insights into disease mechanisms and establish a foundation for therapeutic development.

### Therapeutic development

Therapeutic development builds upon SC-based disease models by evaluating candidate interventions before clinical testing. Traditional discovery workflows have employed high-throughput and high-content screening, target-based assays, and sequential experimental validation to identify promising compounds and assess toxicity [98]. ML complements these approaches by prioritizing candidate compounds, integrating heterogeneous biological and clinical data, reducing experimental burden, and quantitatively predicting therapeutic efficacy, safety, and treatment response.

The reviewed applications encompass major stages of the preclinical therapeutic pipeline. Transcriptomic studies support CSC abundance and potency profiling, drug repurposing, and viral-vector safety prediction [45,107,108]. Morphological and functional studies enable toxicity screening, pharmacological-response prediction, and graft tracking [44,109–115]. Clinical evidence synthesis further supports comparative evaluation of regenerative therapies through Bayesian network meta-analysis [50,116–119]. Collectively, these applications demonstrate the growing role of ML in integrating transcriptomic, morphological, biochemical, functional, and clinical information to improve candidate selection, reduce experimental burden, and support evidence-based therapeutic development.

Reported performance varied according to therapeutic objectives, data modalities, and study design rather than the intrinsic capability of ML methods. Functional pharmacological-response prediction achieved accuracies of approximately 72% to 84% across contractility, calcium-transient, and electrophysiological assays [44,109,110,113,114]. Deep-learning-based toxicity screening achieved accuracies of 87% to 97% for nephrotoxicity and cardiotoxicity assessment [111,115], highlighting the effectiveness of image-based phenotyping for automated high-throughput toxicity screening. ML-based graft tracking and quantification also demonstrated excellent predictive performance (*R*^2^: 0.990), supporting noninvasive monitoring of transplanted SC-derived organoids [112]. Gene-expression-based viral-vector safety prediction achieved an AUC of 0.940 with 90.9% accuracy [108], while transcriptomic CSC profiling achieved 98.67% accuracy and an F1-score of 0.980 [45], demonstrating the utility of ML for therapeutic target characterization and candidate prioritization. In addition, ML-guided drug repurposing identified 5 of 6 experimentally validated candidate compounds capable of inducing CSC differentiation [107], illustrating the potential of ML-guided computational prioritization to accelerate therapeutic discovery.

Validation strategies primarily relied on cross-validation, internal holdout evaluation, and independent experimental validation, whereas independent external validation across multiple datasets or institutions remained relatively uncommon. Clinical evidence synthesis studies further demonstrated the value of Bayesian network meta-analysis for comparing therapeutic efficacy and safety across multiple interventions [116,118,119]. These findings highlight the potential of ML to accelerate therapeutic development by integrating efficacy, toxicity, pharmacological response, and clinical evidence, thereby supporting more efficient, data-driven, and precision-guided translation of candidate therapies.

### Clinical decision support

Clinical decision support represents the final stage of translation, where biological knowledge is combined with patient-level information to support individualized medical decisions. Unlike the preceding application areas, clinical decision support integrates heterogeneous real-world clinical data with SC-derived biological data. Building upon traditional statistical risk models, ML enables integrated analysis of complementary molecular, imaging, biochemical, clinical, and multimodal data to support individualized prediction.

Clinical decision-support applications span 5 major SC data types. Transcriptomic and epigenomic studies use gene expression profiling to identify transcriptional signatures associated with clinical outcomes [120]. Morphology-based studies apply bone-marrow microscopy to predict posttransplant relapse risk [33]. Biochemical and functional studies use plasma protein expression profiling to predict treatment response following mesenchymal SC therapy [121]. Clinical data constitute the largest application area, supporting donor selection, granulocyte-colony-stimulating-factor-induced SC mobilization, transplantation planning, therapeutic drug monitoring, and prediction of posttransplant mortality, relapse, and survival [31,122–128]. Multimodal studies further integrate clinical variables with germline genomic data to predict posttransplant cognitive impairment [129].

The reviewed applications predominantly focus on transplantation medicine, particularly hematopoietic SC transplantation and hematopoietic cell transplantation. Overall, these studies demonstrate how heterogeneous clinical and SC-derived biological data support patient stratification, individualized risk prediction, treatment optimization, and clinical decision-making across the transplantation continuum.

Predictive performance varied according to the clinical endpoint, available patient information, and cohort heterogeneity. Registry-based mortality prediction achieved more modest discrimination (AUC ≈ 0.700) [124], whereas donor availability and SC mobilization models achieved higher AUCs (0.830 to 0.890) [122,123]. Posttransplant survival models reported AUCs up to 0.92 or accuracies exceeding 93% in well-defined cohorts [127,128], while plasma protein expression profiling for mesenchymal SC treatment-response prediction achieved an AUC of 0.767 [121]. Imaging- and transcriptomic-based studies similarly demonstrated promising predictive performance (AUC up to 0.800; accuracy up to 88%) [33,120], whereas multimodal models integrating clinical variables with germline single-nucleotide polymorphism genotyping achieved an AUC of 0.710 for predicting posttransplant cognitive impairment [129]. These performance differences primarily reflect variation in clinical endpoints, patient populations, available data, and validation strategies rather than inherent superiority of one ML approach over another.

Clinical implementation requires substantially stronger evidence than experimental applications because models must demonstrate reliable performance across diverse patient populations and healthcare settings before routine clinical adoption. Although several studies incorporated independent external cohorts, prospective multicenter validation remains uncommon and represents an important prerequisite for routine clinical implementation. Consequently, clinical decision support represents the most demanding stage of ML translation, requiring robust external validation, prospective evaluation, and demonstration of clinical utility before routine deployment.

## Discussion and Future Directions

### ML advancing SC research

Three overarching trends emerge from the reviewed literature. First, ML has become an integral component of the SC research continuum, extending from SC biology and engineering to disease modeling, therapeutic development, and clinical decision support. Rather than replacing experimental investigations, ML complements conventional biological approaches by enabling automated analysis of increasingly complex molecular, morphological, functional, and clinical data while uncovering patterns that are difficult to identify using traditional analytical methods.

A second major trend is the increasing alignment between SC data characteristics, biological questions, and ML model design. Recent advances in transformer-based and foundation models, together with multimodal learning, indicate a broader shift toward pretrained representations, transferable learning, and integration of heterogeneous SC data. As larger standardized datasets become available, these approaches are expected to complement existing ML methods by improving representation learning, cross-dataset generalization, and multimodal analysis. Nevertheless, successful applications will continue to depend on selecting computational approaches that appropriately match the underlying data characteristics, biological objectives, and practical constraints.

A third major trend is the increasing emphasis on robust model evaluation as research progresses from biological discovery toward clinical translation. Exploratory studies often rely on internal validation because of limited data availability, whereas clinically oriented applications increasingly require evidence from independent external cohorts and, ultimately, prospective evaluation before deployment in healthcare settings.

### Limitations

#### Limitations of the current literature

Despite rapid progress, several challenges continue to limit the development and translation of ML in SC research. Many studies remain based on relatively small, laboratory-specific datasets that differ substantially in donor characteristics, SC lines, experimental protocols, culture conditions, and data acquisition procedures. Such biological and technical heterogeneity complicates direct comparison across studies and limits model robustness and transferability. In addition, publicly accessible benchmark datasets remain scarce, reporting practices are inconsistent, and independent evaluation across external datasets or biological cohorts is still limited, particularly for microscopy, functional measurements, and laboratory-generated datasets. Together, these issues hinder reproducibility, objective comparison of ML methods, and translation across laboratories and clinical settings. Future progress will require standardized experimental protocols, comprehensive metadata, publicly accessible benchmark datasets, multicenter collaborations, and shared evaluation frameworks.

#### Limitations of this review

This review has several limitations. First, because both ML and SC research are rapidly evolving fields, recently published studies may not have been included. Second, owing to substantial heterogeneity in SC types, experimental designs, data modalities, ML methodologies, evaluation metrics, and validation strategies, this review provides a qualitative synthesis rather than a formal quantitative meta-analysis. Third, studies in Tables 5 and 6 were categorized according to their primary data source and research objective, although many investigations naturally span multiple data modalities and application domains. Finally, the reviewed literature is not uniformly distributed across SC types. In particular, human-induced-pluripotent-SC-derived cardiomyocytes are disproportionately represented, especially in studies of postdifferentiation maturation. This imbalance reflects the current emphasis of the field on cardiac applications and may limit the extent to which the conclusions of this review generalize across all SC lineages and application domains.

### Future research directions

#### Data resources and benchmark datasets

Future progress depends on standardized, publicly accessible SC data resources. Community efforts should prioritize standardized experimental protocols, comprehensive metadata, data-sharing frameworks, and multicenter collaborations that better capture biological variability across donors, SC lines, differentiation stages, disease models, and experimental platforms. Such resources will improve reproducibility, facilitate external validation, and enable objective comparison of ML methods.

Benchmark datasets represent a critical component of this infrastructure. Future benchmarks should integrate biological diversity, high experimental quality, comprehensive metadata, external validation, multimodal measurements, and public accessibility (Table 7). Although no currently available dataset satisfies all of these criteria simultaneously, the representative sequencing and imaging datasets discussed in this review illustrate complementary characteristics that can guide the development of future community benchmark resources.

**Table 7.** Proposed benchmark dataset selection framework illustrated using representative sequential [64] and imaging [76] datasets

Criterion	Key components (purpose)	[64]	[76]
Accessibility	Public availability of datasets, metadata, and source code to ensure reproducibility and transparent benchmarking	√	√
Quality	Large, biologically diverse, well-annotated datasets generated using standardized experimental protocols and preprocessing pipelines to support robust model development	√	√
Validation	Independent evaluation across datasets, biological cohorts, or experimental conditions to assess model robustness and generalizability	∆	√
Extensibility	Support for incorporating additional data modalities, cell types, or future datasets to facilitate evolving machine learning applications	√	∆

√, criterion substantially satisfied; ∆, criterion partially satisfied or supported by limited evidence

#### Model development and clinical translation

Building upon standardized data resources, future research should prioritize the development of accurate, robust, interpretable, computationally efficient, and clinically deployable ML models. Rather than simply developing increasingly complex architectures, future work should systematically evaluate when multimodal learning, self-supervised learning, and foundation models provide meaningful advantages over conventional approaches for specific SC applications. As standardized benchmark datasets become available, rigorous comparisons across model families, together with task-specific fine-tuning and external validation, will be essential for establishing their practical value.

At the same time, explainable artificial intelligence, uncertainty estimation, and model calibration will remain essential for improving biological interpretability, identifying meaningful biomarkers, and increasing confidence in model predictions. Beyond predictive modeling, ML also has considerable potential to support real-time monitoring of SC cultures through computer vision systems capable of detecting abnormal differentiation, contamination, and loss of viability, thereby improving manufacturing quality control. Additional opportunities include optimizing clinical trial design through patient stratification, cohort selection, and adaptive trial monitoring, as well as emerging applications in chemical reprogramming, SC aging, and digital cell modeling.

For clinical translation, methodological advances must be accompanied by prospective multicenter studies, standardized reporting, regulatory evaluation, and independent validation to ensure that ML systems are reliable, reproducible, and suitable for deployment in regenerative medicine and clinical decision support. Adoption of standardized reporting guidelines, transparent model documentation, and reproducible computational workflows will further facilitate independent validation, regulatory evaluation, and clinical adoption.

## Conclusion

This review provides a comprehensive synthesis of ML applications across SC biology, engineering, and clinical translation through a unified framework encompassing SC data types, experimental data sources, data representations, analytical methodologies, and translational applications. By organizing the rapidly expanding literature within a consistent taxonomy and proposing a framework for benchmark dataset development, this review highlights how different ML approaches align with specific biological questions, data modalities, and clinical objectives.

The reviewed literature demonstrates that successful ML applications depend not on a single algorithm, but on appropriate alignment between SC data characteristics, computational methodologies, and biological objectives, together with rigorous validation and biologically meaningful interpretation. Although substantial progress has been achieved, challenges related to biological heterogeneity, data standardization, model generalizability, and clinical validation continue to limit broader adoption.

Future advances will depend on standardized data resources, robust and interpretable ML methods, multimodal learning, foundation models, and rigorous evaluation supported by close collaboration among SC biologists, clinicians, data scientists, and regulatory stakeholders. Together, these developments have the potential to accelerate biological discovery and facilitate the safe and effective translation of SC research into regenerative and precision medicine.
